# LncRNA AB209371 up-regulated Survivin gene by down-regulating miR-203 in ovarian carcinoma

**DOI:** 10.1186/s13048-019-0559-4

**Published:** 2019-10-10

**Authors:** Zi-Hui Zheng, Dong-Mei Wu, Shao-Hua Fan, Xin Wen, Xin-Rui Han, Shan Wang, Yong-Jian Wang, Zi-Feng Zhang, Qun Shan, Meng-Qiu Li, Bin Hu, Yuan-Lin Zheng, Jun Lu

**Affiliations:** 1 State Key Laboratory Cultivation Base For TCM Quality and Efficacy, School of Medicine and Life Sciences, Nanjing University of Chinese Medicine, Nanjing, 210023 People’s Republic of China; 2 Key Laboratory for Biotechnology on Medicinal Plants of Jiangsu Province, School of Life Science; College of Health Sciences, Jiangsu Normal University, No. 101, Shanghai Road, Tongshan District, Xuzhou, 221116 Jiangsu Province, People’s Republic of China; 3 College of Health Sciences, Jiangsu Normal University, Xuzhou, Jiangsu 221116 People’s Republic of China

**Keywords:** AB209371, Ovarian carcinoma, miR-203, Survivin

## Abstract

AB209371 gene has been characterized as an oncogenic lncRNA in liver cancer. However, its involvement in ovarian carcinoma (OC) is unknown. In the present study, we analyzed the roles of AB209371 in OC. We found that AB209371 gene and Survivin gene were up-regulated in OC and positively correlated with OC development. AB209371 over-expression led to up-regulated Survivin in OC cells, while Survivin over-expression failed to affect AB209371. In addition, AB209371 over-expression led to down-regulated miR-203. However, miR-203 over-expression failed to affect AB209371, but down-regulated the expression of Survivin. In addition, over-expressions of AB209371 and Survivin resulted in the increased proliferation rate of OC cells. Over-expression MiR-203 played the opposite role and attenuated the effects of AB209371 over-expression. Therefore, AB209371 may down-regulate miR-203 to up-regulate Survivin, thereby promoting OC cell proliferation. Our study provided novel insights into the pathogenesis of OC.

## Introduction

Ovarian carcinoma (OC) is a widely known female malignancy [1]. In 2018, there are about 22,240 new cases of OC that was diagnosed, and about 14,070 new cases of deaths in the United States [2]. In China, OC is the 4th leading cause of cancer-related death [1]. The high mortality rate o OC is mainly caused by the fact that most OC patients are diagnosed with inoperable disease [3]. This situation is unlikely to be changed in the near future owing to the lack of sensitive and specific markers at early stages [4]. The unclear pathogenesis of OC is the main challenge in the development of novel diagnostic markers and therapeutic targets [5, 6]. Therefore, in-depth investigations of the molecular pathogenesis of OC are needed.

Genetic factors are the most important players in OC [7]. Survivin, also known as BIRC5, or baculoviral inhibitor of apoptosis repeat-containing 5, is a cell apoptosis inhibitor [8]. In cancer biology, Survivin is usually over-expressed. This inhibits cancer cell apoptosis and promotes cancer cell proliferation, and thus accelerates cancer progression [9]. Therefore, inhibition of Survivin is a potential approach to treat cancers [10]. Certain miRNAs, such as miR-203, can target Survivin to inhibit cancer development [11]. In our preliminary transcriptome analysis, we found that the expression level of Survivin was positively correlated with AB209371, which was a characterized oncogenic lncRNA in hepatocellular carcinoma (HCC) [12]. This study aimed to analyze the interactions between Survivin and AB209371 in OC.

## Materials and methods

### OC patients

Sixty one OC patients (epithelial, high-grade serous tumor) were selected from the 105 OC patients who were admitted to School of Life Science, College of Health Sciences, Jiangsu Normal University from May 2016 to January 2019. This study was approved by the review board of Ethics Committee of School of Life Science, College of Health Sciences, Jiangsu Normal University. The inclusion criteria are: 1) newly diagnosed OC cases; 2) no other clinical disorders were diagnosed; 3) no therapies were initiated. And the exclusion criteria are: 1) recurrent OC patients; 2) history of malignancies; 3) therapies were performed within three months before admission. After admission, the patients were informed of the principle of the present study. All of them signed informed consent. According to the system established by AJCC, there were 12, 23, and 26 cases at clinical stage II-IV, respectively.

### OC cells and tissue samples

Before the initiation of any therapies, an ovarian biopsy was performed on each patient to obtain both non-tumor ovarian (within 2 cm around the tumor and contained less than 2% cancer cells) and OC tissues (contained more than 95% cancer cells). All tissues were directly dropped into a liquid nitrogen tank for storage. UWB1.289 (ATCC, USA) human OC cell line was used. A mixture of 50% MEGM medium and 50% RPMI-1640 Medium (3% FBS) was used to cultivate cells. Cell culture conditions were: 37 °C, 95% humidity, and 5% CO_2_.

### Transient transfections

AB209371 and Survivin expression vectors were constructed using by inserting AB209371 and Survivin cDNAs into the pcDNA3.1 vector (RIBOBIO, Guangzhou, China). Negative control (NC) miRNA (5′-GGUUCUCGGAAUCGUACGAGCUAGC-3′) and miR-203 mimic (5′-AGUGGUUCUUAACAGUUCAACAGUU-3′) were synthesized by RIBOBIO. UWB1.289 cells were harvested at the confluence of 70–80%, followed by lipofectamine 2000 (RIBOBIO) to transfect 35 nM miRNA (NC miRNA as NC group) or 10 nM vector (empty vector as NC group) into 5 × 10^5^ cells in each well of a 6-well plate. Cells were all harvested at 24 h post-transfection to perform subsequent experiments. Control (C) cells in all groups were un-transfected cells.

### RNA extractions and qPCRs

UWB1.289 cells were harvested at 24 h after all transfections and counted. OC and non-tumor tissues were ground in liquid nitrogen. Total RNAs in these cells and tissue samples were extracted using RNAzol (Sigma-Aldrich, USA). It is noted that 85% ethanol was used to precipitate RNAs to harvest miRNAs.

After RNA extractions, all RNA samples were digested for 1 h at 37 °C with DNase I to remove genomic DNA. Following that, reverse transcriptions were performed using AMV Reverse Transcriptase (Promega Corporation) with total RNAs as a template. To measure the expression levels of AB209371 and Survivin mRNA, QuantiTect SYBR Green PCR Kit (Qiagen) was used to prepare all qPCR reaction mixtures with GAPDH as an endogenous control.

To measure the expression levels of miR-203, 3′-polyadenylation of mature miRNAs, reverse transcriptions, and qPCR assays were all performed using All-in-One™ miRNA qRT-PCR Detection Kit (Genecopoeia). All steps were carried out according to the instructions from Genecopoeia. U6 was used as the endogenous control. All experiments were performed with three replicates and analyzed by the 2^-ΔΔCT^ method.

### Cell proliferation assay

CCK-8 kit (Sigma-Aldrich, USA) was used to analyze the effects of different transfections on the proliferation of UWB1.289 cells. Briefly, UWB1.289 cells were collected at 24 h post-transfection, and the numbers were counted. Following that, 3 × 10^4^ UWB1.289 cells were mixture with 1 ml cell above culture medium (3% FBS) to make cell suspensions. The cell was cultured using a 96-well plate (100 μl in each well) at 37 °C. 10 μl CCK-8 solution was added into each well to monitor cell proliferation at 2 h before the termination of cell culture. After the end of cell culture, each well was supplemented with 10 μl DMSO, followed by the measurement of OD values (450 nm).

### Protein extractions and western blot

UWB1.289 cells were collected at 24 h post-transfection, and numbers were counted. Following that, total proteins in 5 × 10^5^ UWB1.289 cells were extracted using RIPA solution (Sangon, USA). After denaturing for 5 min in boiling water, electrophoresis was performed to separate different proteins using 10% SDS-PAGE gel. After that, proteins were transferred to PVDF membranes. Then the membrane was blocked for 2 h in PBS containing 5% non-fat milk at room temperature. Rabbit Survivin (1: 1200, ab469, Abcam) and GAPDH (1: 1000, ab37168, Abcam) primary antibodies were used to incubate the membranes for 15 h at 4 °C, followed by incubating by anti-goat IgG- HRP (1:1000; ab6721; Abcam) for 2 h at 24 °C. ECL substrate (ab65623, Abcam) was utilized to develop signals. Image J v1.48 software was used to process all signals.

### Data process

Data used in all statistical analyses were presented as mean values from 3 biological replicates. Differences between OC and non-tumor tissues were analyzed using paired t-test. Differences among different cell transfection and patient groups were examined using one-way ANOVA (Tukey test). Correlations were made by Pearson’s correlation coefficient and linear regression. *P* < 0.05 was statistically significant.

## Results

### AB209371 and Survivin mRNA were up-regulated in OC and affected by clinical stages

**Fig. 1:**
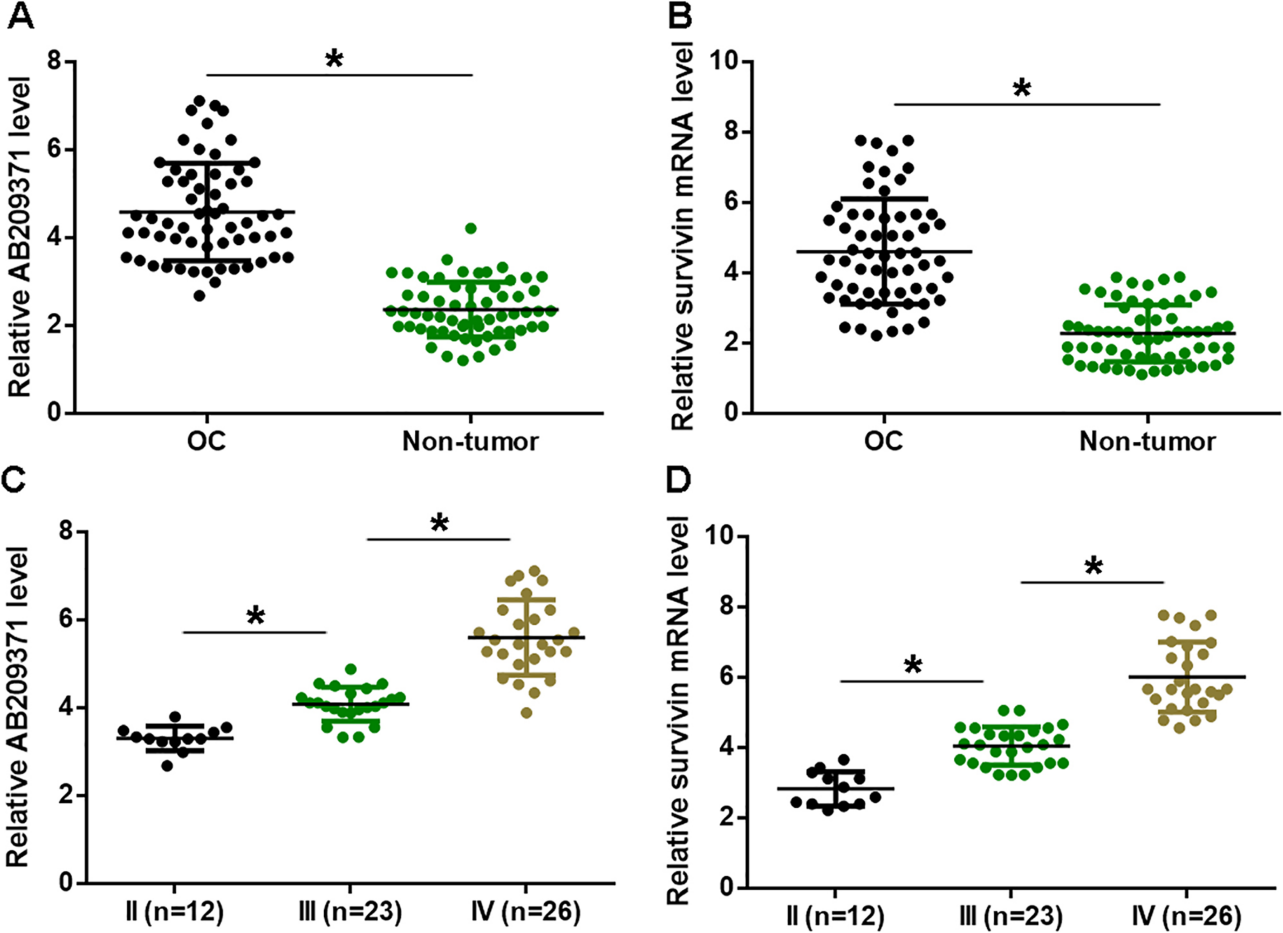
AB209371 and Survivin mRNA were up-regulated in OC and affected by clinical stages. Expression levels of AB209371 (**a**) and Survivin mRNA (**b**) in two types of tissues were measured by qPCR and compared by paired t test. Expression levels of AB209371 (**c**) and Survivin mRNA (**d**) in OC tissues were compared among 3 clinical stages by ANOVA (one-way) and Tukey test. Mean values were presented, *, *p* < 0.05

Expression levels of AB209371 and Survivin mRNA in two types of tissues were measured by qPCR and compared by paired t-test. Comparing to the expression levels in non-tumor tissues, expression levels of AB209371 (Fig. 1a) and Survivin mRNA (Fig. 1b) were significantly higher in non-tumor tissues (*p* < 0.05). According to the system established by AJCC, 61 OC patients had 12, 23, and 26 cases diagnosed with clinical stage II-IV, respectively. Expression levels of AB209371 and Survivin mRNA in OC tissues were compared among three clinical stages by ANOVA (one-way) and Tukey test. It can be observed that mRNA expression levels of AB209371 (Fig. 1c) and Survivin (Fig. 1d) increased significantly by the elevation of clinical stages (*p* < 0.05).

### AB209371 and Survivin mRNA were positively correlated

**Fig. 2:**
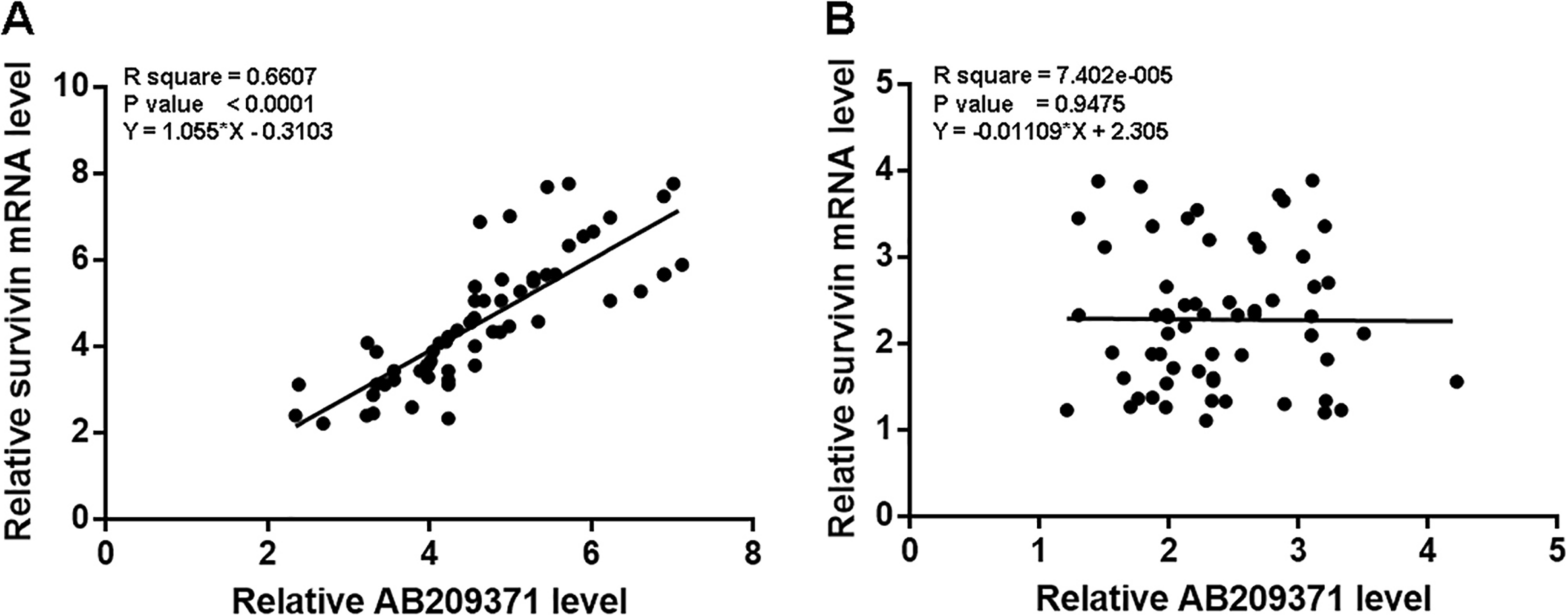
AB209371 and Survivin mRNA were positively correlated. Linear regression was used to analyze the correlations between AB209371 and Survivin mRNA in OC tissues (**a**) and non-tumor tissues (**b**)

Linear regression was used to analyze the correlations between AB209371 and Survivin mRNA. It can be observed that the expression level of AB209371 was significantly and positively correlated with the expression level of Survivin mRNA in OC tissues (Fig. 2a). In contrast, expression levels of AB209371 and Survivin mRNA were not significantly correlated in non-tumor tissues (Fig. 2b).

### AB209371 up-regulate Survivin by down-regulating miR-203

**Fig. 3:**
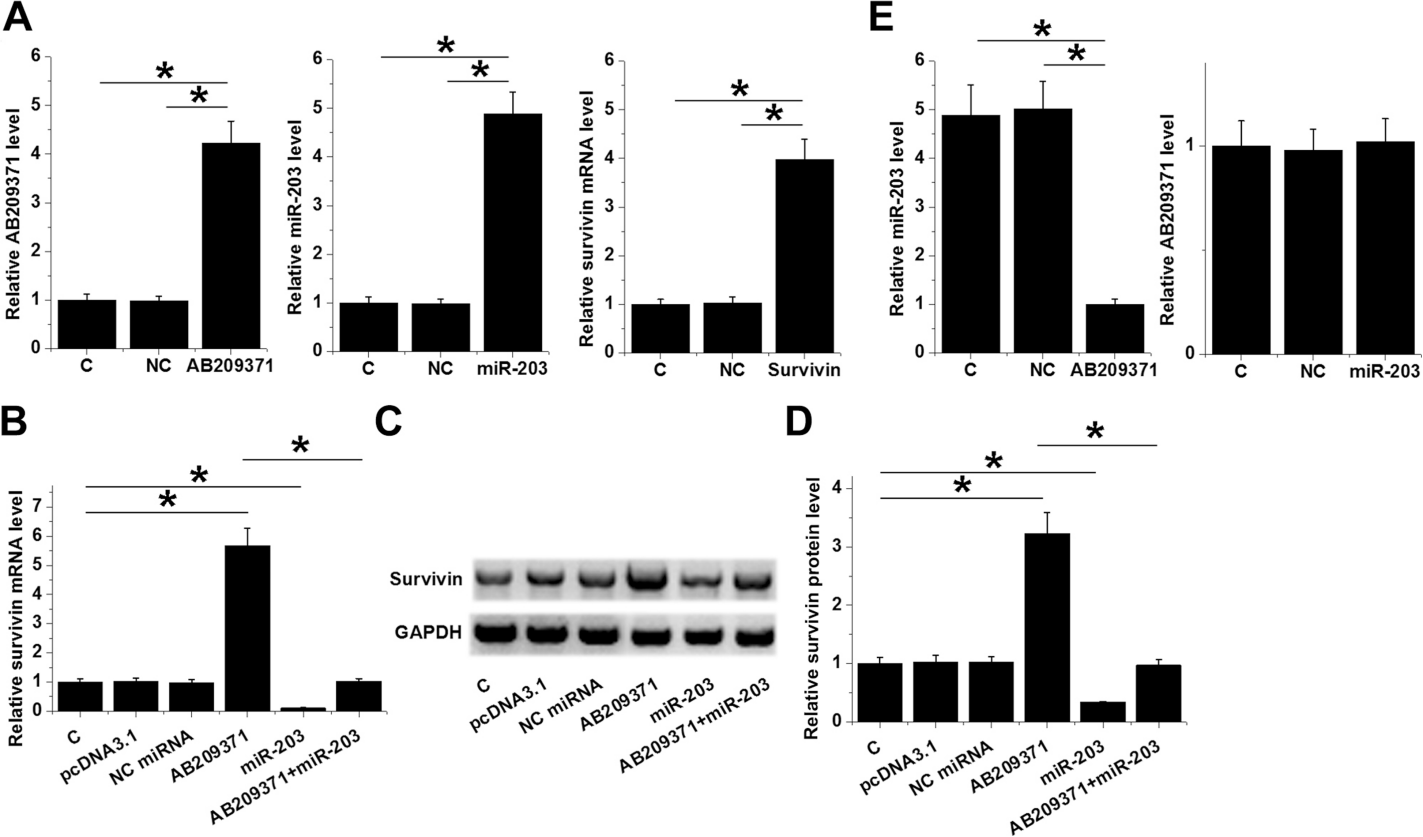
AB209371 up-regulate Survivin by down-regulating miR-203. UWB1.289 cells were transfected with AB209371 and Survivin vectors as well as miR-203 mimic. At 24 h post-transfection, expression levels of AB209371, Survivin mRNA and miR-203 were measured and over-expression was confirmed by qPCR (**a**). Effects of AB209371 and miR-203 over-expression on Survivin mRNA in OC cells were analyzed by qPCR (**b**). Effects of AB209371 and miR-203 over-expression on Survivin protein in OC cells were analyzed by Western blot, and the representative images were showed in (**c**). Quantification data of western blot were shown in (**d**). The interaction between miR-203 and AB209371 was analyzed by qPCR (**e**). Mean values were presented, *, *p* < 0.05

UWB1.289 cells were transfected with AB209371 and Survivin vectors as well as a miR-203 mimic. At 24 h post-transfection, expression levels of AB209371, Survivin mRNA, and miR-203 were measured. Comparing to C and NC groups, expression levels of AB209371, Survivin mRNA, and miR-203 were significantly increased (Fig. 3a, *p* < 0.05). Moreover, AB209371 over-expression led to up-regulated Survivin mRNA, while miR-203 over-expression led to down-regulated Survivin mRNA in OC cells (Fig. 3b, *p* < 0.05). Western blot was performed to evalulate the effects of AB209371 and miR-203 over-expression on the expression of Survivin at protein level, and the representative bands were shown in Fig. 3c. Analysis of the quantification data of western blot showed that, AB209371 over-expression led to up-regulated Survivin protein, while miR-203 over-expression led to down-regulated Survivin protein in OC cells (Fig. 3d, *p* < 0.05). Moreover, miR-203 over-expression attenuated the effects of AB209371 on Survivin expression (Fig. 3e, p < 0.05). In addition, AB209371 over-expression led to down-regulated miR-203, while miR-203 over-expression failed to affect AB209371 but down-regulated Survivin (Fig. 3c, *p* < 0.05).

### AB209371 promoted the proliferation of UWB1.289 cell through miR-203 and Survivin

**Fig. 4:**
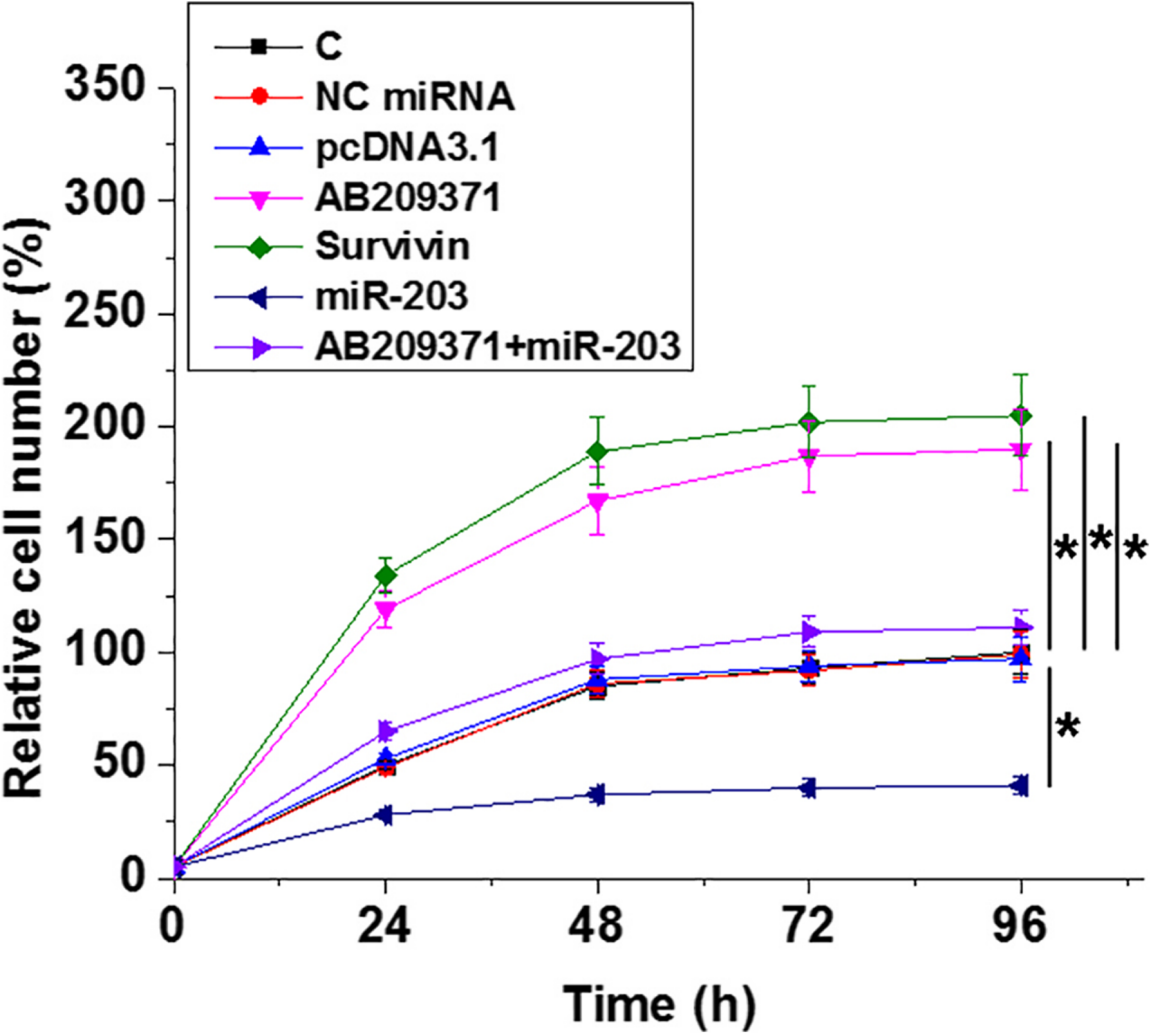
AB209371 promoted the proliferation of UWB1.289 cell through miR-203 and Survivin. The effects of AB209371, Survivin mRNA and miR-203 over-expression on cell proliferation were analyzed by CCK-8 assay. Mean values were presented, *, *p* < 0.05

The effects of AB209371, Survivin mRNA, and miR-203 over-expression on cell proliferation were analyzed by CCK-8 assay. Comparing to NC and C groups, AB209371 and Survivin over-expression resulted in the increased proliferation rate of OC cells. MiR-203 over-expression played the opposite role and attenuated the effects of AB209371 over-expression (Fig. 4, *p* < 0.05).

## Discussion

This study analyzed the roles of AB209371 in OC. We found that AB209371 was up-regulated in OC and promoted the expression of Survivin to accelerate the proliferation of OC cells. The actions of AB209371 are likely to be mediated by miR-203. The expression pattern and functions of AB209371 have only been investigated in HCC [12]. In HCC, AB209371 induced epithelial-mesenchymal transition by activating TGF-β signaling pathway [12]. This study reported the up-regulation of AB209371 in HCC. In addition, over-expression of AB209371 resulted in the increased proliferation rate of HCC cells. Therefore, AB209371 is likely an oncogenic lncRNA in OC.

Survivin is over-expressed in many types of cancers, including OC [13]. Over-expressed Survivin mediated the accelerated proliferation of cancer cells [14]. Consistent with previous studies, our study observed the over-expression of Survivin in OC, and the increased proliferation rate of OC cells after Survivin over-expression. Therefore, our data confirmed the oncogenic role of Survivin in OC. Expression of Survivin in cancer biology can be regulated by many miRNAs. For instance, miR-218 can target Survivin in cervical cancer to inhibit lymph node metastasis [15]. In laryngeal squamous cell carcinoma, miR-34a can target Survivin to inhibit postoperative occurrence [16]. In laryngeal carcinoma, Bian et al. reported that miR-203 targeted Survivin to induce G1 phase cell cycle arrest, thereby inhibiting cancer growth [11]. In the present study, we also observed down-regulated Survivin at both mRNA and protein levels. Therefore, miR-203 may also target Survivin in OC.

We also found that AB209371 may up-regulate Survivin by suppressing miR-203. It is known that TGF-β can inhibit miR-203 in cancers to promote cancer progression [17]. In HCC, AB209371 activated TGF-β signaling pathway [12]. Therefore, TGF-β may mediate the down-regulation of miR-203 induced by AB209371 over-expression. Our future studies will investigate the involvement of TGF-β in this process.

In conclusion, AB209371 is over-expressed in OC and may up-regulate Survivin by down-regulating miR-203, which in turn inhibits cancer cell proliferation.

## Data Availability

The analyzed data sets generated during the study are available from the corresponding author on reasonable request.
